# A241 AMOXICILLIN-CLAVULANATE INDUCED LIVER INJURY WITH CONCOMITANT AMPULLARY STENOSIS: A CASE REPORT AND LITERATURE REVIEW

**DOI:** 10.1093/jcag/gwae059.241

**Published:** 2025-02-10

**Authors:** K Kecskemeti, N Gibbings, S Fung

**Affiliations:** Faculty of Medicine, University of Toronto, Toronto, ON, Canada; Faculty of Medicine, University of Toronto, Toronto, ON, Canada; Faculty of Medicine, University of Toronto, Toronto, ON, Canada

## Abstract

**Background:**

Amoxicillin-clavulanate drug-induced liver injury (DILI) has been reported at a rate of 1 in 2,400 prescriptions^1^. Patients tend to present with a cholestatic pattern of liver enzyme elevation while hepatocellular enzyme elevation is more commonly reported in younger patients. In mild cases, once amoxicillin-clavulanate is discontinued the patient’s recovery tends to be rapid.

**Aims:**

We present a patient case and a literature review regarding concomitant ampullary stenosis and amoxicillin-clavulanate induced liver injury.

**Methods:**

We present a case report outlining a patient’s course and management in hospital. We then conducted a literature review on Pub-Med using the following terms: “ampulla stenosis” and “drug-induced liver injury”.

**Results:**

A 56-year-old female with a remote history of a cholecystectomy was prescribed amoxicillin-clavulanate for suspected diverticulitis. She completed a 5-day course and then represented with worsening abdominal pain. She was found to have significant transaminitis with a mixed cholestatic-hepatocellular pattern (ALT 726, AST 351, ALP 289). She underwent an MRCP which showed extensive extrahepatic and intrahepatic biliary duct dilations which was disproportionate to expected post-cholecystectomy changes and suggestive of ampullary stenosis. Amoxicillin-clavulanate was discontinued on admission and her liver enzymes normalized as an out-patient three weeks after admission (ALT 34, ALP 125). She was referred for outpatient endoscopic ultrasound for assessment and treatment of her ampullary stenosis.

The literature review on PubMed yielded a single case report (n = 1) of a patient with atorvastatin induced liver injury and underlying ampullary stenosis^2^. In that case, the patient improved when the medication was discontinued and then underwent a sphincterotomy.

**Conclusions:**

Although seldomly reported in the medical literature there is one other case report of concomitant drug-induced liver injury in a patient with ampullary stenosis. To our knowledge, this is the first case report of amoxicillin-clavulanate induced liver injury in a patient with ampullary stenosis. Potential mechanisms may include ampullary stenosis related biliary stasis which may exacerbate the toxic effects of medications on the liver.

Patient’s Biochemical Investigations in Hospital

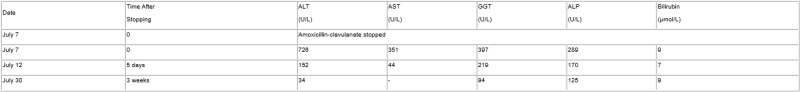

Note: AST was not measured on July 30th. INR was not recorded during the patient’s hospitalization

**Funding Agencies:**

NoneN/A

